# 

**Published:** 1890-08-23

**Authors:** 


SATURDAY, AUGUST 23rd, 1890.

**The Fairy Tales of Science** - 305

**Words of Consolation.**—Care - 306

**An Interview with Mrs. Dacre Craven** (with portrait) 307

**The Doctor's Pulpit.**—The Expansion of Medicine - 309

**Thoughts on Ill-Health.** II.—Audi Alteram Partem - 310

**Everybody's Page** - 311

**Notes and News** - 312

**Annotations.**—An Honourable Shame—Professor Huxley on Doctor's Latin—Cyclists and Rupture - 314

**General Charities** - 318

**The Practitioner's Mirror and Retrospect:**—

The Metropolitan Workhouse Infirmaries—Resolution of the Paddington Board of Guardians - 315

Medicine—Dr. Bevan Rake on Leprosy - 317

Surgery—Gastrotomy - 318

New Drugs, Appliances, and Things Medical—Bovril—Florador - 318

**Students' Column**—University of London - 319

**Scraps and Gleanings** - 320

**Passing Topics** - 320

### EXTRA SUPPLEMENT—THE NURSING MIRROR.

**En Passant**—Another Institution—Zenana Medical College—The Australian Commission—Nurses at Rome—Male Nurses—Nurses' Home, Johannesburg - lxxxvii
**Sketches of Insanity.** VII.—Dementia - lxxxviii
**A Prayer for the Hospital Ward** - lxxxviii
**The Training of Nurses in France** - lxxxix
**A Daring Outrage** - lxxxix
**Everybody's Opinion.** — Workhouse Nurses, lxxxix;—Paris Institutions—Intemperate Nurses - xc
**Death in our Ranks** - xc
**Notes and Queries—Vacancies** - xc
**Amusements and Relaxation** - xc

